# Deriving Ligand Orientation in Weak Protein–Ligand Complexes by DEEP‐STD NMR Spectroscopy in the Absence of Protein Chemical‐Shift Assignment

**DOI:** 10.1002/cbic.201800568

**Published:** 2018-12-13

**Authors:** Ridvan Nepravishta, Samuel Walpole, Louise Tailford, Nathalie Juge, Jesus Angulo

**Affiliations:** ^1^ School of Pharmacy University of East Anglia Norwich Research Park Norwich Norfolk NR4 7TJ UK; ^2^ The Gut Microbes and Health Institute Strategic Program Quadram Institute of Bioscience Norwich Research Park Norwich Norfolk NR4 7UA UK

**Keywords:** DEEP-STD, mixed molecular dynamics, NMR spectroscopy, TEMPOL

## Abstract

Differential epitope mapping saturation transfer difference (DEEP‐STD) NMR spectroscopy is a recently developed powerful approach for elucidating the structure and pharmacophore of weak protein–ligand interactions, as it reports key information on the orientation of the ligand and the architecture of the binding pocket.^1^ The method relies on selective saturation of protein residues in the binding site and the generation of a differential epitope map by observing the ligand, which depicts the nature of the protein residues making contact with the ligand in the bound state. Selective saturation requires knowledge of the chemical‐shift assignment of the protein residues, which can be obtained either experimentally by NMR spectroscopy or predicted from 3D structures. Herein, we propose a simple experimental procedure to expand the DEEP‐STD NMR methodology to protein–ligand cases in which the spectral assignment of the protein is not available. This is achieved by experimentally identifying the chemical shifts of the residues present in binding hot‐spots on the surface of the receptor protein by using 2D NMR experiments combined with a paramagnetic probe.

The 3D structure of a small bioactive molecule in complex with its receptor gives atomic information that is essential for understanding the biological effects triggered by biomolecular recognition processes, as well as for the discovery and design of new drugs. Several techniques are used to achieve this aim, with X‐ray crystallography, NMR spectroscopy and, more recently, cryo‐electron microscopy being the most relevant approaches.

Recently, we developed the DiffErential EPitope mapping saturation transfer difference (DEEP‐STD) NMR methodology for weak protein–ligand interactions,^1^ as an extension of the general STD NMR method.^2^ The DEEP‐STD NMR technique allows the orientation of the ligand to be derived through differential selective saturation of different sets of key protein residues in the binding site. Namely, two STD NMR experiments are carried out, each one saturating different sets of protein residues, and the difference between the resulting spectra is quantified and mapped onto the ligand structure (differential epitope map). In order to perform the DEEP‐STD NMR experiment accurately, it is of paramount importance to know beforehand the chemical shifts of the residues present in the binding site, in order to identify which set of residues to target, that is, choosing the irradiation frequencies to ensure that the selective saturation is applied on residues that are present in the binding site. Experimental chemical shifts can be obtained by NMR spectroscopy or derived from a 3D structure obtained by X‐ray diffraction or by homology modelling.^3^ The experimental DEEP‐STD factors can be further combined with molecular docking and STD intensity predictions by CORCEMA‐ST^4^ in order to select the docking model that best fits the experimental data.

For when there is no chemical‐shift assignment of the receptor protein, we here propose a general approach to experimentally identify the chemical shifts of those binding pocket resonances that relies on identifying ligand binding hot‐spots on the surface of the protein (a ligand‐binding hot‐spot is a site on the surface of the protein that has a high probability for interaction with a ligand^5^) by using 2D NMR spectroscopy. This approach is compatible with the STD NMR technique, inexpensive and relatively fast, all of which which should allow broad applicability.

The slow molecular tumbling of large proteins in solution is characterized by an overall correlation time expected to be in the range of 10^−8^ seconds. However, the internal correlation time of the surface residues of globular proteins might be significantly shorter. As a result, residues in the core of the protein follow a slow‐motion regime due to their low flexibility; this causes the signals to become broadened beyond detection. Conversely, the greater flexibility of surface residues causes them to follow a fast‐motion regime that leads to crosspeaks in 2D NMR experiments that will be narrower and, hence, detectable. Therefore, these spectra are more likely to display signals from residues exposed on the surface or in very flexible regions of the protein.^6^

By using 2D ^1^H,^1^H TOCSY experiments, hot‐spots can be readily mapped by adding paramagnetic probes such as 4‐hydroxy‐2,2,6,6‐tetramethylpiperidin‐1‐oxyl (TEMPOL) to a protein sample.^7^ A decrease in the intensity of specific protein TOCSY crosspeaks, compared with the spectrum recorded without the paramagnetic probe, allows those residues interacting with the probe to be easily identified because they are affected by the paramagnetic relaxation enhancement (PRE) effect.^8^, ^9^ Previous PRE studies with TEMPOL have demonstrated the greater accessibility of this probe to proteins′ specific binding sites rather than surface regions.^7^, ^10^, ^11^ As a result of these experiments, the identified hot‐spot resonances can then be considered as input frequencies for the DEEP‐STD NMR experiments.

Binding hot‐spots on the surface of proteins have previously been identified by NMR spectroscopy using paramagnetic probes along with classical molecular dynamics (MD) as well as by mixed MD using 5–50 % probe/water mixtures.^7^, ^12^, ^13^ For the development of our protocol, we combined this approach with DEEP‐STD NMR using the structurally characterized catalytic domain (belonging to glycoside hydrolase family 33, GH33) of the intramolecular *trans*‐sialidase (IT‐sialidase) from the human gut symbiont *Ruminococcus gnavus*, *Rg*NanH‐GH33, in complex with 2,7‐anhydro‐Neu5Ac (PDB ID: 4X4A) as a benchmark.^14^ *Rg*NanH‐GH33 is a 489‐residue domain that can be considered out of the typical range for swift assignment and structure determination by NMR spectroscopy and was previously used to develop the DEEP‐STD NMR approach.^1^

We first performed 2D homonuclear ^1^H,^1^H TOCSY experiments on *Rg*NanH‐GH33. The protein was exchanged in 10 mm [D_11_]Tris D_2_O buffer (pH 7.8) with 100 mm NaCl and used at a concentration of 1.2 mm. First, a 2D ^1^H,^1^H TOCSY reference spectrum of the protein was acquired, then two spectra in the presence of 2 and 12 mm of TEMPOL. The spectra obtained in the absence or in the presence of increasing concentrations of TEMPOL showed that the probe selectively interacts with some residues of the protein, as only some resonances in the spectra were significantly affected, as seen by a decreased intensity (Figure 1). The chemical shifts most affected by the presence of TEMPOL were at 0.6, 0.74, 1.06, 1.15, 1.26, 6.6, 6.74, 7.04, 7.57, 8.56 ppm (Figure S1 in the Supporting Information). These resonances, although lacking a specific assignment, are typical of aliphatic and aromatic amino acids, and we can exclude the presence of the NH resonances in the spectra as the protein was solvated in a D_2_O buffer. The identified resonances from the TEMPOL‐attenuated TOCSY spectra of *Rg*NanH‐GH33 were indeed in very good agreement with the predicted chemical shifts of key aliphatic and aromatic residues in the binding pocket of the enzyme (Ile258, Ile338, Val502, Thr557, Tyr525, Tyr677 and Trp698).^1^

**Figure 1:**
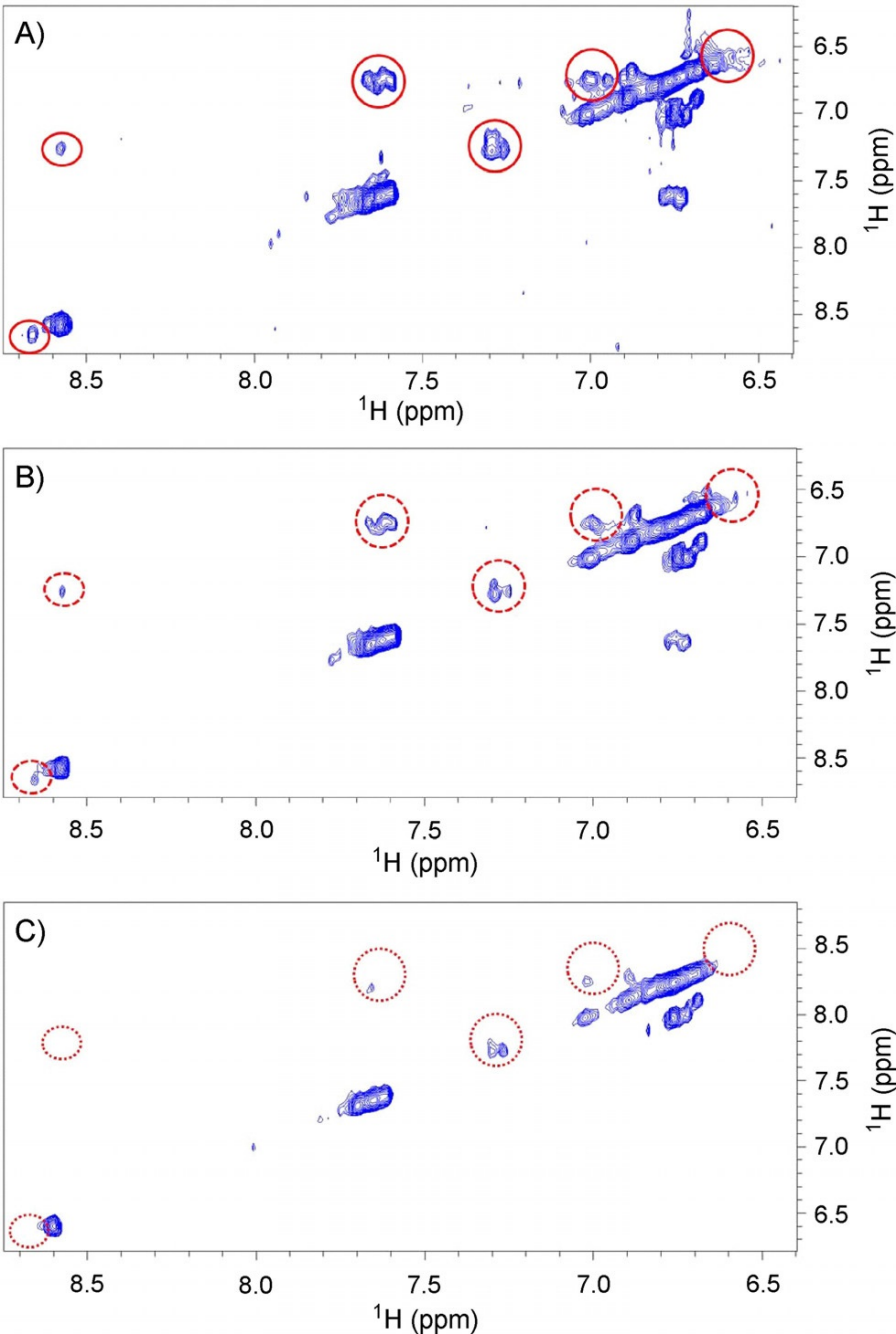
Expansion of the aromatic spectral region of the TOCSY spectra of 1.2 mm *Rg*NanH‐GH33 A) alone or in the presence of B) 2 or C) 12 mm TEMPOL. Red circles highlight some resonances affected by the presence of TEMPOL (see also Figure S1).

To further validate our approach and exclude false positives (i.e., binding hot‐spots outside the binding site), we carried out MD simulations, an approach successfully used in the past to identify ligand binding pockets for the development of small‐molecule inhibitors.^15^ Here, MD simulations were used to confirm the accessibility of TEMPOL to the specific binding pocket of *Rg*NanH‐GH33. To efficiently explore the configurational space of the *Rg*NanH‐GH33‐TEMPOL system, three different MD approaches were considered: 1) long MD (1.0 μs) with a low concentration of TEMPOL (10 mm) starting from a random configuration of the system, 2) 16 independent replicas of short MD (0.8 μs, total 10 mm of TEMPOL), and 3) 16 independent short replica MD simulations with a high concentration (50 % *w*/*w*) of TEMPOL in water, known as the MixMD approach.^12^

In each case, we first analysed the backbone RMSD of *Rg*NanH‐GH33 for each trajectory, and showed that the presence of TEMPOL did not affect the structure of the protein, even for MixMD, as the average backbone RMSD was only approximately 1 Å (Figure S5). In the case of the long MD and the 16 short replicas, for which there were relatively few molecules of TEMPOL in the simulation, the interaction between TEMPOL and *Rg*NanH‐GH33 was analysed by computing the contacts between TEMPOL and each residue in *Rg*NanH‐GH33 over the course of each trajectory, in order to construct a fractional occupancy map for each residue. In the case of MixMD, in which there were 381 molecules of TEMPOL in the bounding box, the occupancy was measured by using a 0.5 Å grid to create bins for each TEMPOL molecule in each frame of the trajectory. The resulting 3D histograms were then visualized by means of the isomesh feature in PyMOL^16^ by using a structure averaged over the whole trajectory for *Rg*NanH‐GH33 (Figure 2).

**Figure 2:**
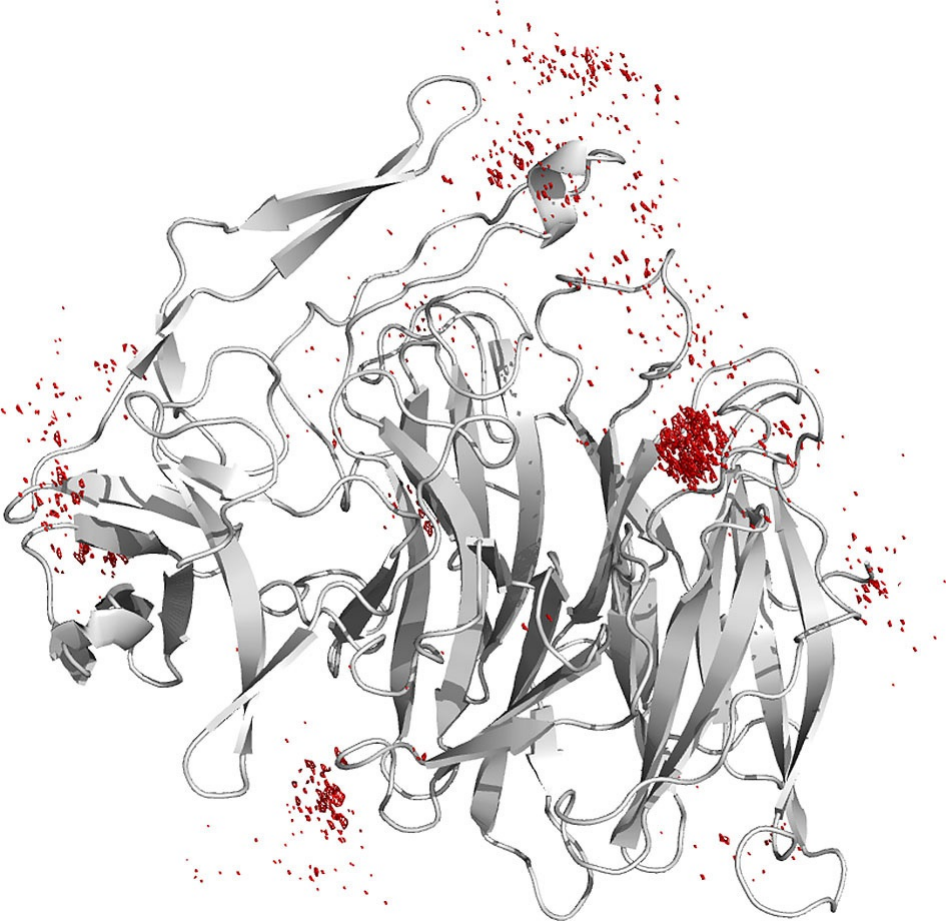
Distribution of TEMPOL (red) around *Rg*NanH‐GH33 (grey) as determined by MixMD. The distribution reveals a single large hot‐spot at the known 2,7‐anhydro‐Neu5Ac binding site, thereby revealing that TEMPOL can act as a probe to selectively target residues of the binding site.

Firstly, the long MD simulation containing a low concentration of TEMPOL did map several binding hot‐spots, including the area of the binding site, but the outcome was dependent on the starting coordinates of the system. To overcome this issue, the same experiment was repeated with 16 different, independent short replicas of 50 ns each, according to a previous protocol.^7^ In this case, although the mapping of the binding hot‐spots was clear, the extension of sampling of the surface was not complete (Figure S5). In the MixMD approach, high concentrations of the probe enabled most of the protein surface to be mapped in a short time. In order to avoid biasing the system by the starting coordinates, 12 independent trajectories were run starting from different initial random configurations of the system. Figure 2 displays the average structure of the protein together with the occupancy grid, showing that the area of the known binding site of the 2,7‐anhydro‐Neu5Ac ligand is the major site for the interaction with the paramagnetic probe.

This result clearly excludes the presence of false positives and, more importantly, confirms that TEMPOL is selective for the binding site. Together with the TEMPOL/*Rg*NanH‐GH33 interaction TOCSY experiment, these MD data build a solid argument for the use of TEMPOL‐based TOCSY experiments to identify specific chemical shifts from residues in the binding pocket in order to perform the DEEP‐STD NMR experiments.

We then carried out the DEEP‐STD NMR study with the frequencies identified by the TEMPOL approach. *Rg*NanH‐GH33 (50 μm) in the presence of 2,7‐anhydro‐Neu5Ac (1 mm) in 10 mm [D_11_]Tris D_2_O buffer (pH 7.8) with 100 mm NaCl at 298 K was saturated with a train of Gaussian pulses of 50 ms each for 0.75 s, centred on the chemical shifts of the binding hot‐spots 0.6, 0.74, 1.06, 1.15, 1.26, 6.6, 6.74, 7.04, 7.57, 8.56 ppm.

As the absence of chemical‐shift assignment of the protein prevents the irradiation frequencies to specific protons in the binding pocket from being known, we propose here a novel approach: instead of using a single pair of frequencies to determine the differential epitope map of the ligand (DEEP‐STD map),^1^ an averaging approach should be followed. First, the DEEP‐STD factors for each experiment resulting from all the possible pairs of aliphatic and aromatic frequencies experimentally identified before are calculated (Figure S3). In our case, this resulted in 25 differential epitope maps. Secondly, all the obtained DEEP‐STD factors are averaged to obtain a unique DEEP‐STD map. This approach produces a more accurate depiction of the orientation and the nature of the amino acids surrounding the ligand in the binding pocket, particularly when no chemical shifts from the protein are available.

Figure 3 A shows the experimental average DEEP‐STD map of 2,7‐anhydro‐Neu5Ac binding to *Rg*NanH‐GH33.

**Figure 3:**
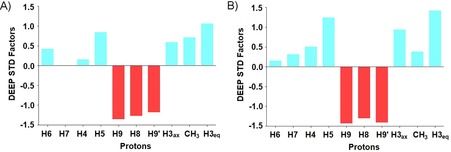
A) Experimental average DEEP‐STD factors of the binding of 2,7‐anhydro‐Neu5Ac to *Rg*NanH‐GH33 obtained from 25 differential epitope maps through the use of selective saturation at pairs of frequencies from the two sets experimentally determined by the TOCSY+TEMPOL experimental approach; set 1 (0.6, 0.74, 1.06, 1.15, 1.26 ppm) and set 2 (6.6, 6.74, 7.04, 7.57, 8.56 ppm; Figure S3). B) Theoretical average DEEP‐STD factors calculated by using CORCEMA‐ST with ranges of saturation frequencies encompassing the experimental values in the calculation.

The map highlights that CH_3,_ H3_ax_, H3_eq_, H5 are oriented toward aliphatic residue protons, whereas H6, H7, H4 present little to no preferred orientation, and H9, H9′ and H8 protons are oriented toward aromatic residues. This result is in excellent agreement with the crystal structure of the complex between 2,7‐anhydro‐Neu5Ac and *Rg*NanH‐GH33.^1^ To confirm that our average DEEP‐STD map is a reliable representation of the architecture of the binding pocket, we compared theoretical predictions of the average DEEP‐STD map by using the CORCEMA‐ST approach (see the Supporting Information).^4^ The average DEEP‐STD factors calculated by using CORCEMA‐ST (Figure 3 B) are in excellent agreement with the experimentally obtained ones. This result further validates our approach, demonstrating that the TEMPOL‐based TOCSY approach is a reliable and powerful approach for identifying the suitable set of saturating frequencies to carry out DEEP‐STD NMR studies in the absence of protein chemical‐shift assignment.

Although here we have applied this approach to an enzyme with a polar binding pocket that favours H‐bond interactions with TEMPOL, it has been previously shown that the interaction between proteins and TEMPOL can involve weak van der Waals forces, hydrogen bonding and hydrophobic interactions. Several authors have described interactions of TEMPOL with proteins such as ubiquitin, lysozyme, tendamistat, Sso7d, cyclophyllin, and BTPI,^10^, ^17^, ^18^, ^19^, ^20^, ^21^ which present different hydrophobicity/hydrophilicity profiles in their binding sites; this makes us confident of the general applicability of the new protocol to different types of protein target. Nonetheless, some expected limitations are that TEMPOL must bind the protein with low affinity so as to allow an easy interpretation of the spectra in the absence or in the presence of the paramagnetic agent, and it should not induce changes in the conformation of the protein upon binding, which would lead to misinterpretation of resonances to consider for DEEP‐STD NMR or to conformational instability of the protein. The competition of TEMPOL with water tightly bound to the protein is also worth noting; in unfavourable cases this might prevent the probe from approaching the protein surface.^18^

In summary, we have developed a simple experimental procedure to expand the field of application of the DEEP‐STD NMR methodology for deriving ligand orientation to protein–ligand cases in where the spectral assignment of the protein is not available, that is, when 1) a full NMR assignment is not possible, 2) the predicted chemical shifts from the structure are not in line with the experimental data (e.g., due to the dynamics of the protein, not accounted for in calculations on a static X‐ray structure) or 3) chemical‐shift assignments are lacking. Combining 2D TOCSY experiments in the absence/presence of a paramagnetic probe with the determination of an average DEEP‐STD map by saturation at all the experimentally determined frequencies has been demonstrated to be a powerful approach to determine the type of protein residues most likely to interact with the ligand. The obtained information on the orientation of the ligand in the binding pocket of the protein opens several interesting applications of the DEEP‐STD NMR methodology, for example in the hit‐to‐lead stage of drug discovery as in 3D‐QSAR studies. Further, if combined with the *K*_D_ of the complex, the experimentally obtained averaged DEEP‐STD factors could be used as descriptors to evaluate success or failure of hit modifications during the hit‐to‐lead stage.

## Conflict of interest

*The authors declare no conflict of interest*.

## Biographical Information

Jesús Angulo obtained his doctorate at the Instituto de Investigaciones Químicas (CSIC/University of Seville) in 2002 under the supervision of Dr. Pedro Nieto. He moved to the group of Prof. Thomas Peters (University of Lübeck, Germany) to specialize in ligand‐based NMR spectroscopy. He rejoined the IIQ in 2008 before joining the School of Pharmacy at the University of East Anglia in 2013, where he is a Senior Lecturer in NMR Spectroscopy. His research group focuses on structural and dynamics characterization of biologically active molecules and their complexes, with a particular focus on glycans, by using NMR spectroscopy and computational techniques.

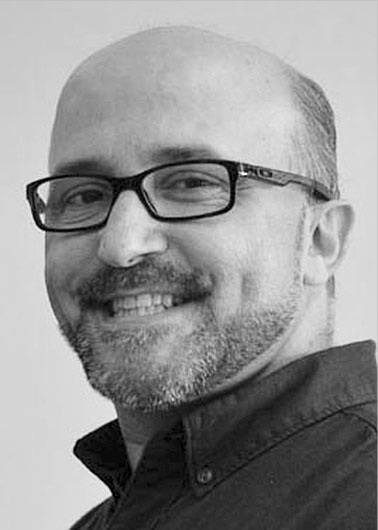

## Supporting information

As a service to our authors and readers, this journal provides supporting information supplied by the authors. Such materials are peer reviewed and may be re‐organized for online delivery, but are not copy‐edited or typeset. Technical support issues arising from supporting information (other than missing files) should be addressed to the authors.

Supplementary
