# Supplementary material for: Deriving Ligand Orientation in Weak Protein–Ligand Complexes by DEEP‐STD NMR Spectroscopy in the Absence of Protein Chemical‐Shift Assignment
Source: Chembiochem. 2018 Dec 13;20(3):340–4. doi: 10.1002/cbic.201800568 (PMC6468252; doi:10.1002/cbic.201800568)
Supplement: Supplementary file 1 — Supplementary [file CBIC-20-340-s001.pdf]

CHEMBIOCHEM

Supporting Information

Deriving Ligand Orientation in Weak Protein–Ligand Complexes by DEEP-STD NMR Spectroscopy in the Absence of Protein Chemical-Shift Assignment

Ridvan Nepravishta+,[a] Samuel Walpole+,[a] Louise Tailford,[b] Nathalie Juge,[b] and Jesus Angulo*[a]

cbic_201800568_sm_miscellaneous_information.pdf

Material and Methods

*Rg*NanH-GH33 was expressed in *E. coli* BL21 and purified by immobilized metal anion chromatography (IMAC) as reported in [1] while 2,7-anhydro-Neu5Ac was prepared as previously described.[2] Deuterium oxide (99.9% ^{2}H), tris-(hydroxymethyl-d$_3$)amino-d$_2$-methane (Tris-d$_{11}$, 98% ^{2}H) and TEMPOL (4-hydroxy-2,2,6,6-tetramethylpiperidine-N-oxyl) were purchased from Sigma.

NMR experiments

All the NMR experiments were conducted in 10 mM Tris-d$_{11}$ D_2O buffer pH 7.8 and 100 mM NaCl at 298 K. For the hot-spot mapping experiment of *Rg*NanH-GH33 with TEMPOL, the protein was concentrated at 1.2 mM using Amicon filters with a 30 kDa cut-off. Three consecutive 2D ^{1}H-^{1}H TOCSY experiments were acquired in the absence and in the presence of 2 mM and 12 mM of TEMPOL, respectively. The ^{1}H-^{1}H TOCSY[3] NMR spectra were acquired on an 800.23 MHz Avance III Bruker spectrometer at 298 K with an SW of 12 ppm acquiring 32 scans per experiment with a TD of 2K data points in the direct dimension and 256 experiments in the indirect dimension. The residual water was suppressed by using the WATERGATE technique.[4,5] The recovery time for the experiment was 2 s while the mixing time was set typically at 0.1 s. 1D ^{1}H STD NMR[6] spectra were acquired with SW of 16 ppm using a TD of 32K data points and a recycling delay (D1) of 3 s with 64 scans. The Bruker library pulse *stdiffesgp.3* was used for all the STD NMR experiments applying a train of 50 ms Gaussian shaped pulse (0.1 mW [40 dB]) at 40 ppm (off-resonance spectra) and 0.6, 0.74, 1.06, 1.15, 1.26, 6.6, 6.74, 7.04, 7.57, 8.56 ppm (on-resonance spectra), to get the averaged differential epitope (DEEP-STD NMR). For all the experiments, a saturation time of 0.75 s was used. The water signal was suppressed by the excitation sculpting technique[7] while the protein signals were removed by using a 40 ms T1ρ filter.

Calculations of DEEP-STD NMR factors

In DEEP-STD NMR,[8] two STD NMR experiments are performed on the same sample (**exp1** and **exp2**), each differing only in the frequency of the saturating pulse. For example, **exp1** may irradiate aromatic groups (7 ppm) and **exp2** may irradiate aliphatic groups (1 ppm). For each proton, i, in the spectrum the DEEP STD NMR factor (**ΔSTD_i**) can be calculated by taking the ratio between the STD intensities of that proton in **exp1** (**$STD_{exp1,i}$**) and in **exp2** (**$STD_{exp2,i}$**) and subtracting from it the <u>average ratio between the two experiments</u>:

$$\Delta STD_i = \frac{STD_{exp1,i}}{STD_{exp2,i}} - \frac{1}{n}\sum_{i}^{n}\left(\frac{STD_{exp1,i}}{STD_{exp2,i}}\right)$$

As different irradiation or experimental conditions can lead to significant changes in the total saturation generated on the protein receptor, an hence in the final STD intensities, to obtain a consistent scale of **ΔSTD_i** factors, <u>**exp1** must be the experiment showing larger total ligand saturation</u>. Using this definition, protons that are unaffected by the change in experimental conditions have **ΔSTD_i** close to zero, whereas **positive values of ΔSTD_i indicate proximity to the groups irradiated in exp1** and **negative values of ΔSTD_i indicate proximity to the groups irradiated in exp2**. In the example above, positive values of ΔSTD_i would indicate proximity to aromatic groups, and negative values of ΔSTD_i indicate proximity to aliphatic groups.

Averaged experimental DEEP-STD NMR processing protocol.

In this work, we slightly modified the previously published DEEP-STD processing protocol[8] by introducing the concept of "**averaged DEEP-STD maps"**, which are obtained from a series of different saturating frequencies (*i.e.*, the set of frequencies derived from the 2D ^{1}H,^{1}H-TOCSY TEMPOL experiments). In this work, 25 individual DEEP-STD maps were obtained. They were:

DEEP-STD maps 1-5:

Fig. S3a) Aliphatic resonance 0.6 ppm, different aromatic resonances (0.6/6.6, 0.6/6.74, 0.6/7.04, 0.6/7.57, 0.6/8.56)

DEEP-STD maps 5-10:

Fig. S3b) Aliphatic resonance 0.74 ppm and different aromatic resonances (0.74/6.6, 0.74/6.74, 0.74/7.04, 0.74/7.57, 0.74/ 8.56)

DEEP-STD maps 10-15:

Fig. S3c) Aliphatic resonance 1.06 ppm and different aromatic resonances (1.06/6.6, 1.06/6.74, 1.06/7.04, 1.06/7.57, 1.06/ 8.56)

DEEP-STD maps 15-20:

Fig. S3d) Aliphatic resonance 1.15 ppm and different aromatic resonances (1.15/6.6, 1.15/6.74, 1.15/7.04, 1.15/7.57, 1.15/ 8.56)

DEEP-STD maps 20-25:

Fig. S3e) Aliphatic resonance 1.26 ppm and different aromatic resonances (1.26/6.6, 1.26/6.74, 1.26/7.04, 1.26/7.57, 1.26/ 8.56)

After that they were averaged for each proton to give a unique profile as a combination of all the frequencies. (See Fig. 3a in the main text). A simple averaging of the DEEP-STD factors for each ligand proton was performed. The rationale behind averaging of the DEEP-STD factors is to consider the global (accumulated) saturation effect that any ligand proton is experiencing from different residue regions around it that are selectively saturated in different experiments when moving the saturation frequency. That is, when only a single frequency is used for, for example, aromatic irradiation, it might be that, under the experimental conditions, the aromatic region surrounding the ligand in the binding pocket is not well saturated, reducing the "quality" of the differential information that can be gained by the DEEP-STD NMR methodology. This can be improved by saturating at different frequencies around that spectral region (e.g., 6.60, 6.74, 7.04, 7.57, 8.56 ppm, at the aromatic spectral region, Fig. S3), and then calculating the average value. Additionally, in this way, we are also taking into consideration the dynamics of ligand ligands protons in the bound state which, in the timeframe of STD measurement, might visit slightly different sites and have slightly different orientations. Calculating the average leads to the best representation of the most populated binding orientation.

Averaged theoretical DEEP-STD data.

In order to evaluate the experimental results, CORCEMA-ST was used to calculate theoretical NMR-STD intensities following similar protocols already published.[9] Briefly the calculations were performed using the X-Ray structure of the RgNanH-GH33 protein in complex with 2,7-anhydro-sialic acid (PDB code 4x4a). The saturation regions for the protein (irrad option in the inputdata.m in CORCEMA-ST) considered for the calculation were 0.0-0.6 ppm; 0-0.75 ppm; 0-1.0 ppm for the aliphatic regions and 5.6-7.5 ppm; 5.5-8.0 ppm for aromatic using values predicted from shiftx2. The outputs of CORCEMA-ST for each saturation region were first were analyzed for their consistency with the experimental STD-NMR data and further used to calculate the averaged DEEP-STD factors in a

similar fashion to the experimentally derived ones. (See main text **Fig. 3b**). The maps obtained in this case are fewer due to the fact that ranges were used that best matched the experimental intensities (**Fig. S4**). The maps shown in the figure show the best correspondence with the experimental data. After that they were averaged to give a unique profile as a combination of all the frequencies. (See main text **Fig. 3b**)

Molecular Dynamics simulations

The coordinates of *Rg*NanH-GH33 were obtained from RCSB PDB protein code 4X4A and prepared using the Protein Preparation Wizard of Schrodinger's Maestro.[10] All non-protein atoms were removed. Protons were then added to the model, using PROPKA to predict the protonation state of polar sidechains at pH 7.[11] The hydrogen-bonding network was automatically optimized by allowing asparagine, glutamine and histidine sidechains to be flipped. The model was then minimized using the OPLS3[12] force field and a heavy atom convergence threshold of 0.3 Å.

A 3D model of the TEMPOL structure and AMBER-compatible parameters were obtained.[13] For the classical MD simulations, a total of 10 TEMPOL molecules were randomly positioned around the protein. For MixMD, Packmol[14] was used to generate a solvent box of 50% w/w TEMPOL in TIP3P water. The system was then built with the *leap* module of AmberTools [15] using the AMBER ff14SB[16] forcefield for *Rg*NanH-GH33. The protein (and 10 TEMPOL molecules for the classical simulations) was solvated in a truncated octahedral bounding box, set such that the protein was at least 10 Å from the box edge in all places. For the classical simulations, the solvent was TIP3P water, whereas, for the MixMD simulation, the solvent was the 50% w/w TEMPOL mixture in TIP3P water. In all cases, 16 Na^+ ions were added to neutralize the system.

Each system was minimized using the conjugate gradient algorithm, converging on a threshold of 10^{-4} kcal mol^{-1} $Å^{-1}$, first with 20 kcal mol^{-1} $Å^{-2}$ restraints on solute atoms, before repeating with no restraints. The system was slowly heated to 310 K over 500 ps (NVT), before equilibrating the pressure to 1 atm (NPT) over a further 500 ps. In both cases, 20 kcal mol^{-1} A^{-2} restraints were used on solute atoms. These restraints were then slowly released over 800 ps to produce the equilibrated system. In all cases, the SHAKE algorithm was used to restrain all bonds involving hydrogen, allowing for a time step of 2 fs. A Langevin thermostat was used with a collision frequency of 5 ps^{-1} and the barostat used an isotropic Berendsen algorithm with a relaxation time of 1 ps. In all cases, periodic boundary conditions were used, using the particle mesh Ewald to calculate electrostatics. The timestep of 1 fs was required to maintain a stable simulation in the presence of TEMPOL.

In all cases, production dynamics were run using the same parameters described for system equilibration. For the long classical MD simulation, the total simulation time was 1 μs. For the replicas, 16 independent 50 ns simulations were performed by initiating each simulation with different randomly generated velocities. For the MixMD, 12 independent 20 ns simulations were performed.

Supplementary Figures

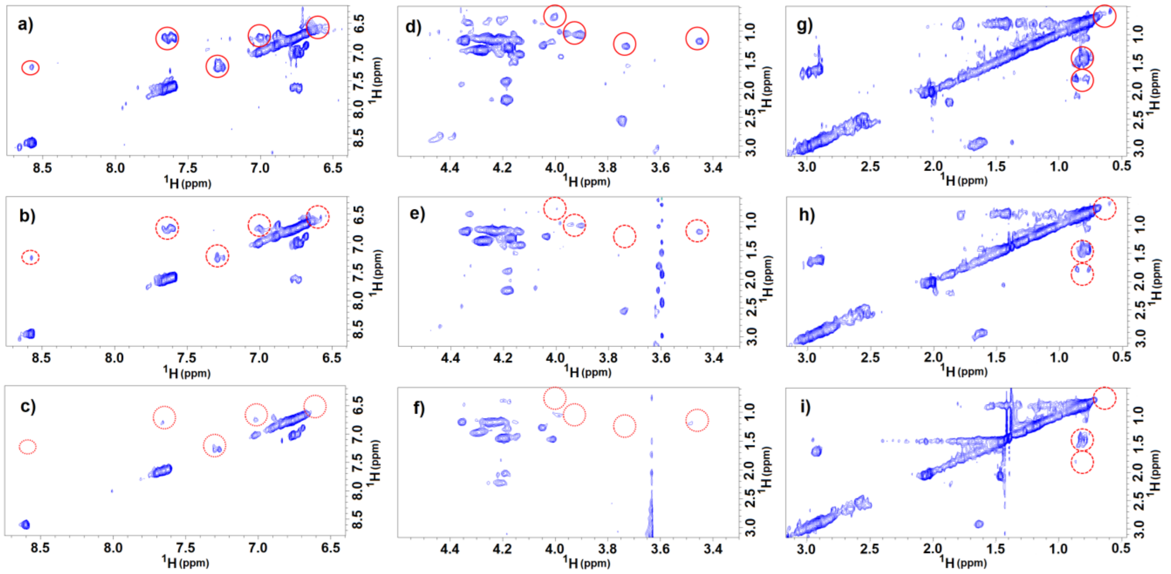

Figure S1. Expansions of **a) aromatic d) Hα** and **g) aliphatic** regions of the ^{1}H-^{1}H TOCSY NMR spectra of *Rg*NanH-GH33 in Tris-d_{11} buffer, at 298 K. In panels **b), e), h),** and **c), f), i)** same regions of the ^{1}H-^{1}H TOCSY spectra in the presence of 2 and 12 mM TEMPOL, respectively. In circles are shown the resonances most affected by TEMPOL (reduced intensities). This frequencies were then used for the STD experiments to carry out the DEEP-STD NMR protocol.

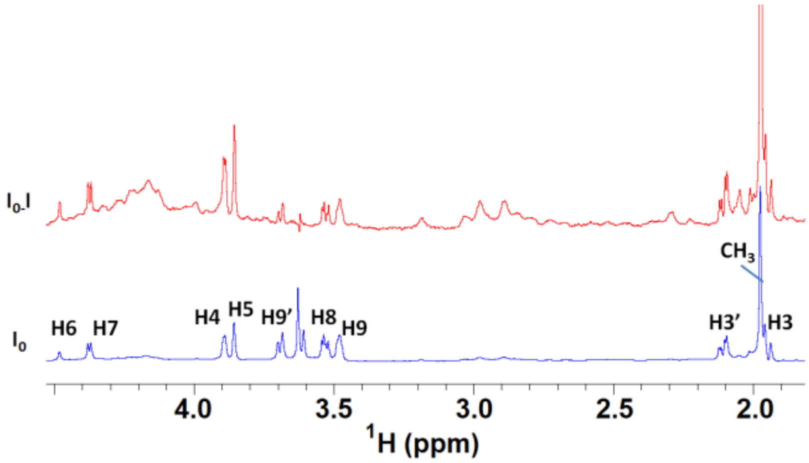

Figure S2. Example of STD NMR of a 1 mM sample of 2,7-anhydro-Neu5Ac in the presence of 50 µM *Rg*NanH-GH33 in deuterated Tris-d_{11} buffer, at 298 K. **I_0**: Reference spectra; **I_0-I**: the STD NMR spectrum irradiated at 0.74 ppm with a saturation time of 0.75 s.

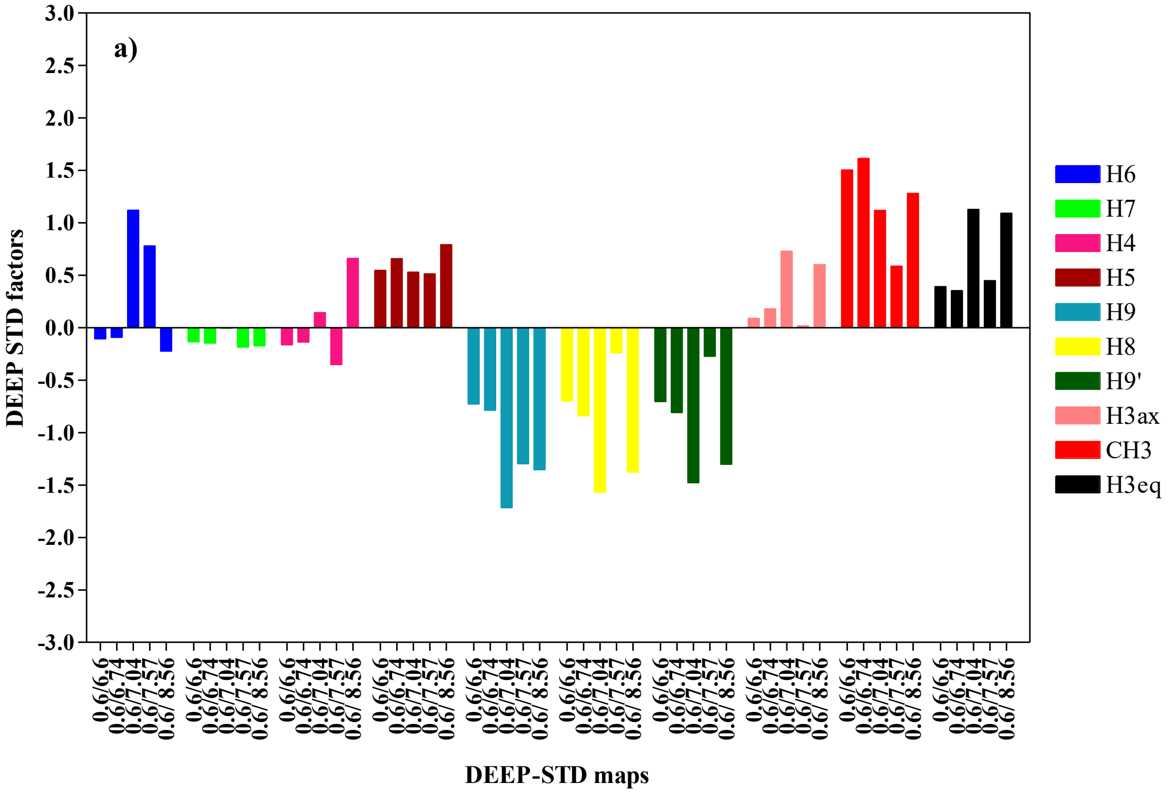

a)
DEEP STD factors
DEEP-STD maps
H6
H7
H4
H5
H9
H8
H9'
H3ax
CH3
H3eq

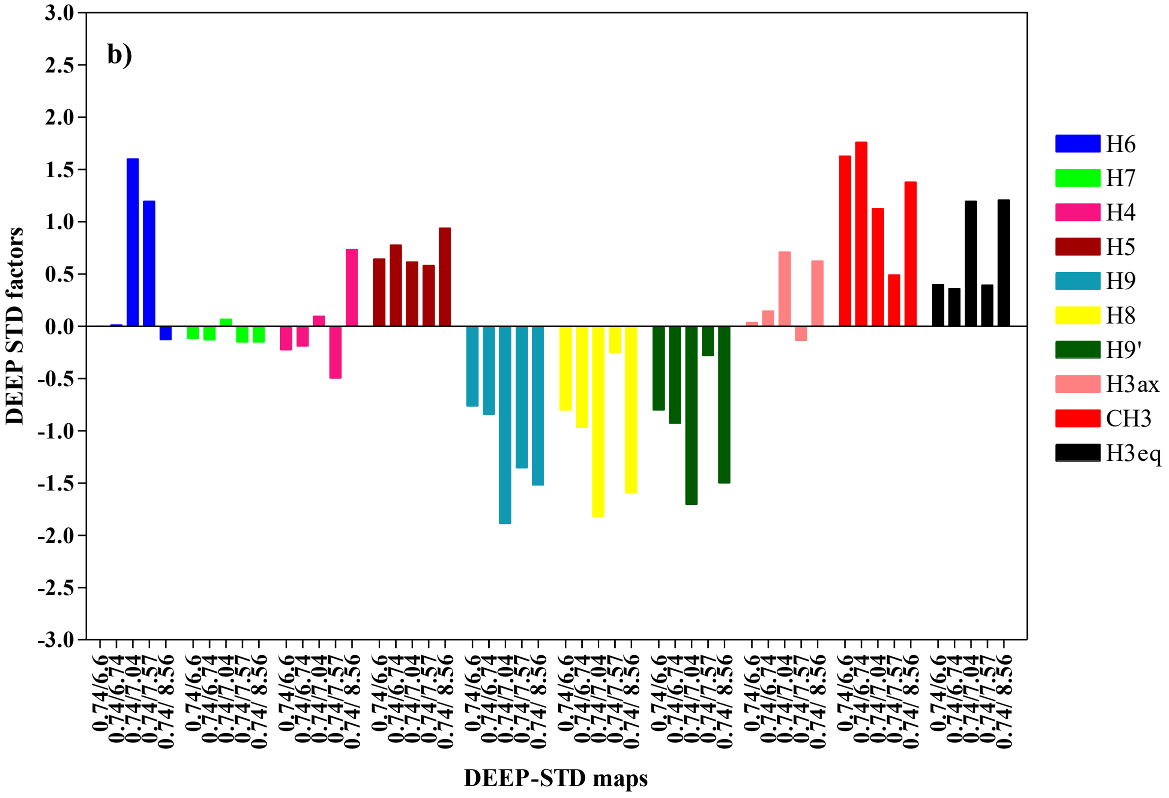

b)
DEEP STD factors
DEEP-STD maps
H6
H7
H4
H5
H9
H8
H9'
H3ax
CH3
H3eq

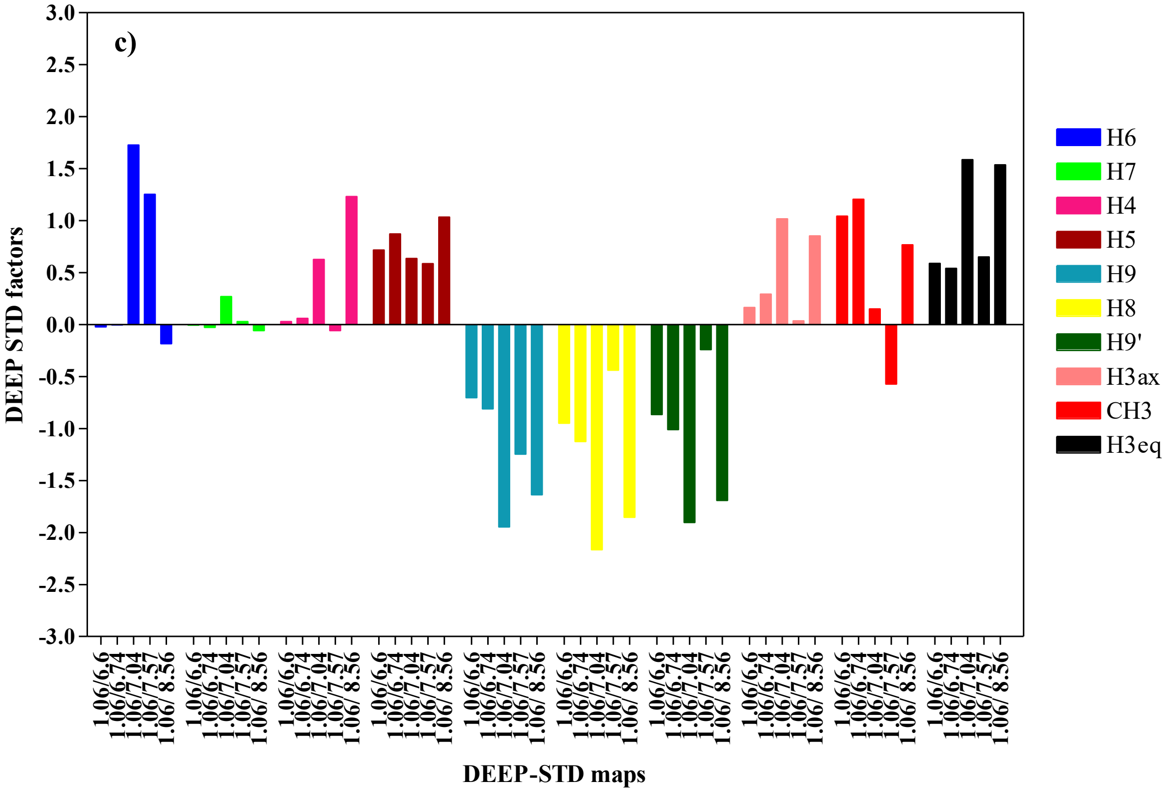
c)
DEEP STD factors
DEEP-STD maps
3.0
2.5
2.0
1.5
1.0
0.5
0.0
-0.5
-1.0
-1.5
-2.0
-2.5
-3.0
1.06/6.6
1.06/6.74
1.06/7.04
1.06/7.57
1.06/ 8.56
H6
H7
H4
H5
H9
H8
H9'
H3ax
CH3
H3eq

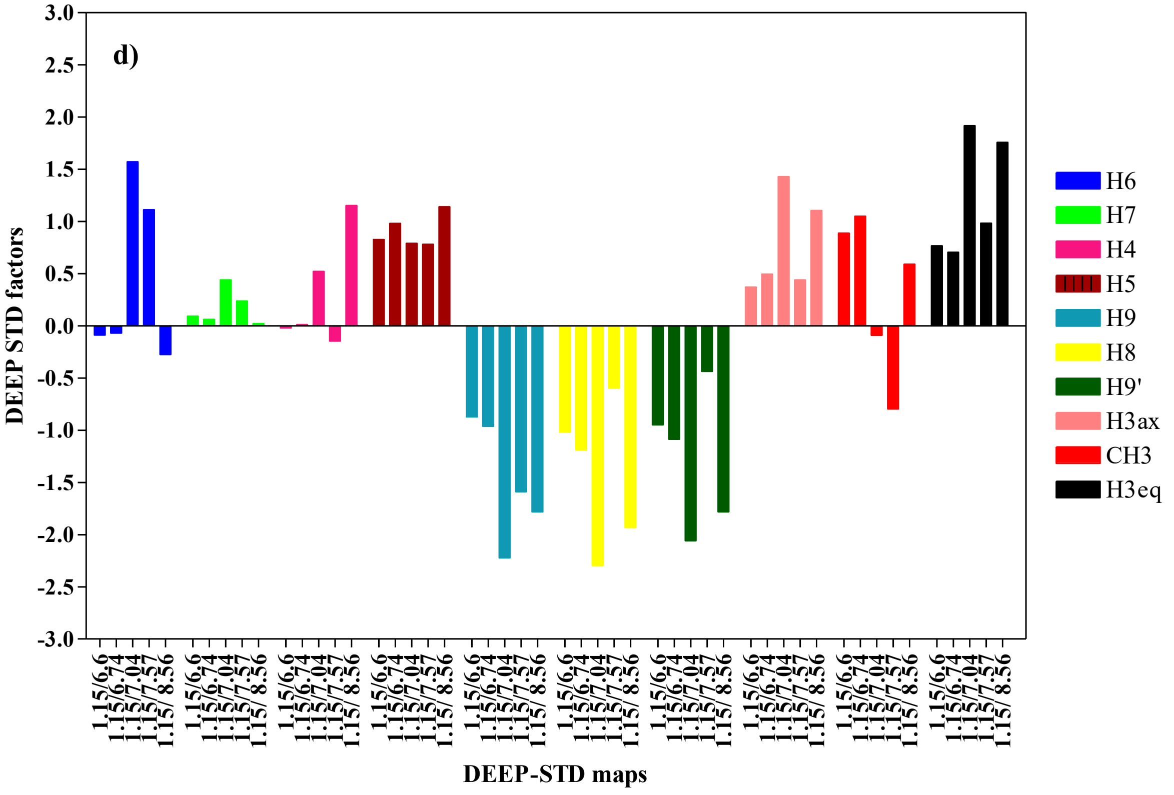
d)
DEEP STD factors
DEEP-STD maps
3.0
2.5
2.0
1.5
1.0
0.5
0.0
-0.5
-1.0
-1.5
-2.0
-2.5
-3.0
1.15/6.6
1.15/6.74
1.15/7.04
1.15/7.57
1.15/ 8.56
H6
H7
H4
H5
H9
H8
H9'
H3ax
CH3
H3eq

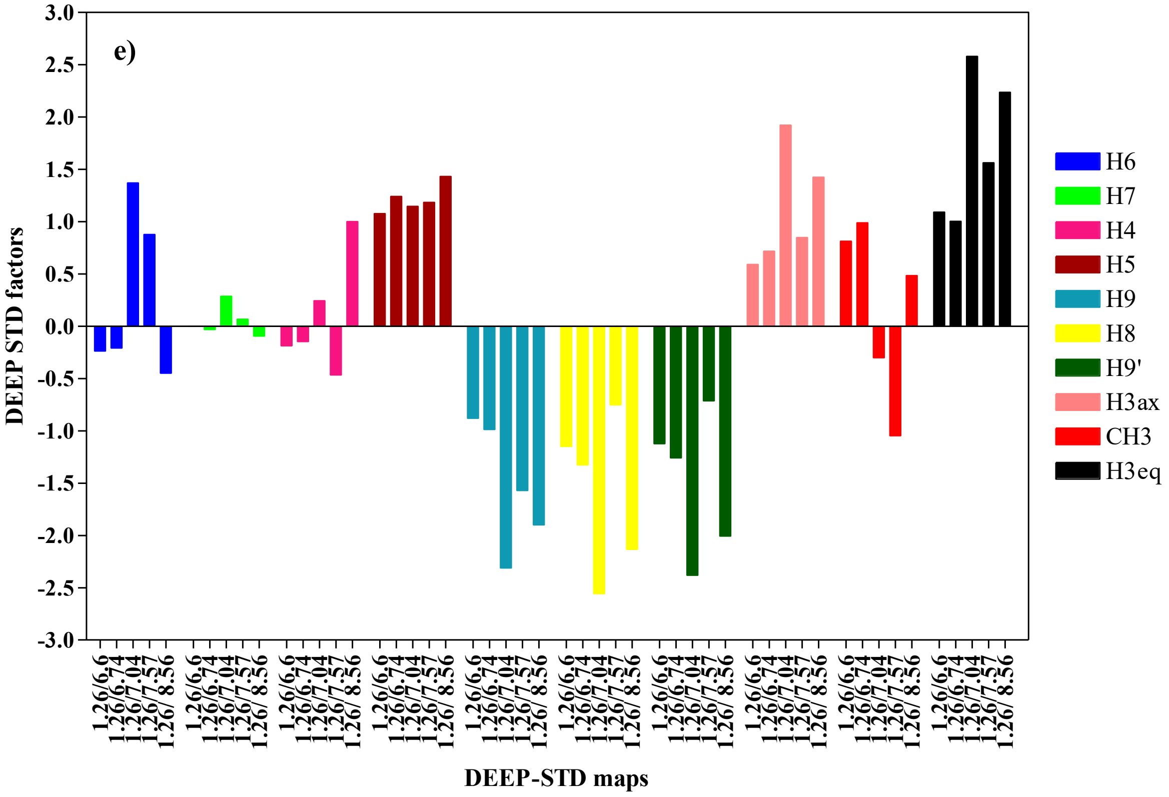

Figure S3. Series of DEEP-STD factors obtained by combining the five aromatic chemical shifts (0.6, 0.74, 1.06, 1.15, 1.26 ppm) and five aliphatic chemical shifts (6.6, 6.74, 7.04, 7.57, 8.56 ppm) identified by the TOCSY+TEMPOL experiments. **a) DEEP-STD maps 1-5** (0.6/6.6, 0.6/6.74, 0.6/7.04, 0.6/7.57, 0.6/8.56), **b) DEEP-STD maps 6-10** (0.74/6.6, 0.74/6.74, 0.74/7.04, 0.74/7.57, 0.74/8.56), **c) DEEP-STD maps 11-15** (1.06/6.6, 1.06/6.74, 1.06/7.04, 1.06/7.57, 1.06/ 8.56), **d) DEEP-STD maps 16-20** (1.15/6.6, 1.15/6.74, 1.15/7.04, 1.15/7.57, 1.15/ 8.56), **e) DEEP-STD maps 21-25** 1.26/6.6, 1.26/6.74, 1.26/7.04, 1.26/7.57, 1.26/8.56). Ligand protons are represented by different colors.

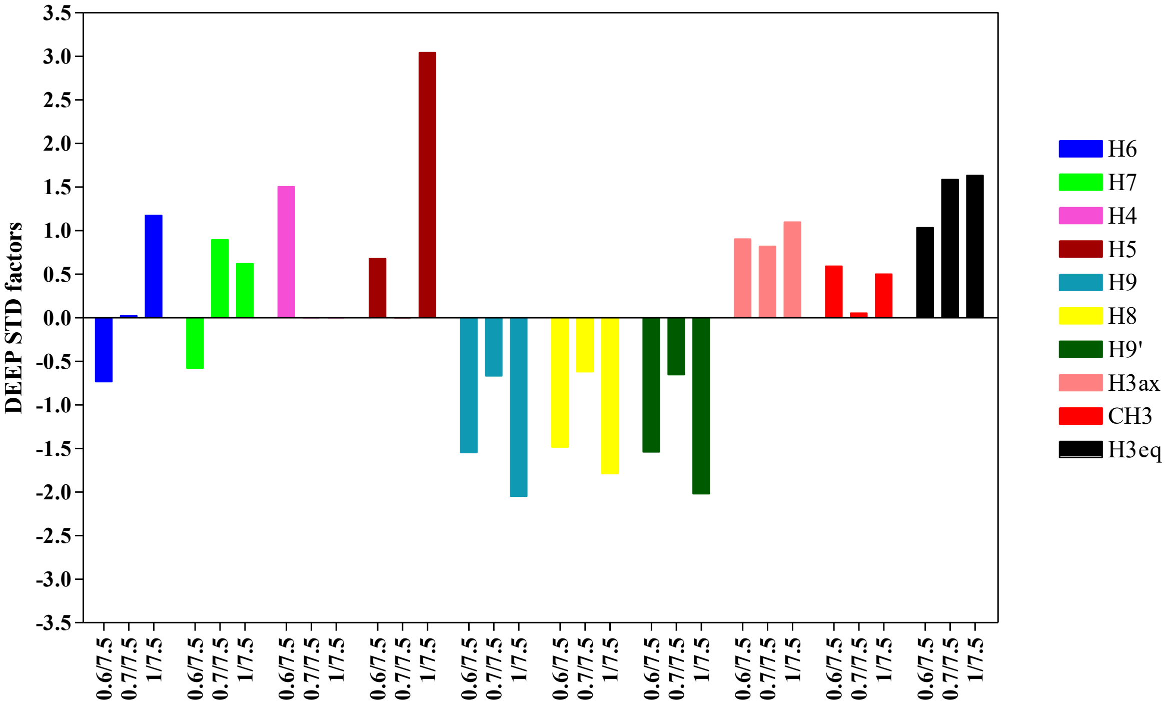

Figure S4. Theoretical DEEP-STD factors calculated with CORCEMA-ST. DEEP-STD factors with differential frequencies 0.6/7.5, 0.7/7.5, and 1.0/7.5. Ligand protons are represented by different colors.

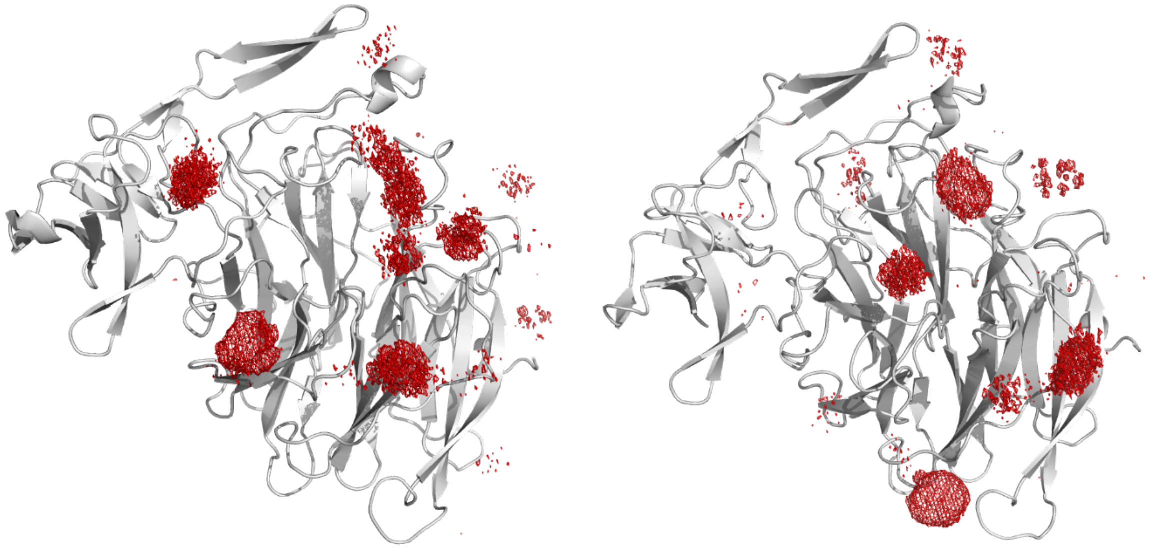

Figure S5. Distribution of the paramagnetic probe TEMPOL (red mesh) around *Rg*NanH-GH33 (grey cartoon) as determined by long classical MD (left) or from 16 short 50 ns independent replicas (right). Both systems contained 10 TEMPOL molecules. Poor sampling resulted in many potential hot-spots being identified (cf. to Fig. 2 in the main text).

References

[1] L. E. Tailford, C. D. Owen, J. Walshaw, E. H. Crost, J. Hardy-Goddard, G. Le Gall, W. M. de Vos, G. L. Taylor, N. Juge, Nat. Commun. 2015, 6:7624, 1-12.

[2] E. H. Crost, L. E. Tailford, M. Monestier, D. Swarbreck, B. Henrissat, L. C. Crossman, N. Juge, Gut Microbes 2016, 7, 302-312.

[3] A. Bax , D.G. Davis, J. Magn. Reson. 1985, 65, 355-360

[4] M. Piotto, V. Saudek & V. Sklenar, J. Biomol. NMR 1992, 2, 661 - 666

[5] V. Sklenar, M. Piotto, R. Leppik & V. Saudek, J. Magn. Reson., 1993 Series A 102, 241 -245

[6] M. Mayer, B. Meyer, Angew. Chem. Int. Ed. 1999, 38, 1784-1788

[7] T.-L. Hwang, A.J. Shaka, J. Magn. Reson. 1995 Series A 112 275-279

[8] S. Monaco, L. E. Tailford, N. Juge, J. Angulo, Angew. Chem. Int. Ed. 2017 ,129 (48), 15491–15495.

[9] P.M. Enríquez-Navas, C. Guzzi, J.C. Muñoz-Garcia, P.M. Nieto, J. Angulo in Methods in Molecular Biology, Glycoinformatics. (Eds.: T. Lütteke, M. Frank), Springer (Humana Press), New-York , 2015, pp. 475-487

[10] Schrödinger Release 2018-1: Maestro, Schrödinger, LLC, New York, NY, 2018

[11] G. M. Sastry, M. Adzhigirey, T. Day, R. Annabhimoju, W. Sherman, J. Comput. Aid. Mol. Des., 2013, 27(3), 221-234

[12] E. Harder, W. Damm, J. Maple, C. Wu, M. Reboul, J.Y. Xiang, L. Wang, D. Lupyan, M. K. Dahlgren, J. L. Knight, J. W. Kaus, D. S. Cerutti, G. Krilov, W. L. Jorgensen, R. Abel, R.A. Friesner, J. Chem. Theory Comput., 2015, 12 (1), pp 281–296

[13] E. Stendardo, A. Pedone, P. Cimino, M. C. Menziani, O. Crescenzi, V. Barone. Phys. Chem. Chem. Phys. 2010, 12, 11697-11709

[14] L. Martínez, R. Andrade, E. G. Birgin, J. M. Martínez. J. Comput. Chem., 2009, 30(13):2157-2164

[15] D.A. Case, I.Y. Ben-Shalom, S.R. Brozell, D.S. Cerutti, T.E. Cheatham, III, V.W.D. Cruzeiro, T.A. Darden, R.E. Duke, D. Ghoreishi, M.K. Gilson, H. Gohlke, A.W. Goetz, D. Greene, R Harris, N. Homeyer, S. Izadi, A. Kovalenko, T. Kurtzman, T.S. Lee, S. LeGrand, P. Li, C. Lin, J. Liu, T. Luchko, R. Luo, D.J. Mermelstein, K.M. Merz, Y. Miao, G. Monard, C. Nguyen, H. Nguyen, I. Omelyan, A. Onufriev, F. Pan, R. Qi, D.R. Roe, A. Roitberg, C. Sagui, S. Schott-Verdugo, J. Shen, C.L. Simmerling, J. Smith, R. Salomon-Ferrer, J. Swails, R.C. Walker, J. Wang, H. Wei, R.M. Wolf, X. Wu, L. Xiao, D.M. York and P.A. Kollman, AMBER 2018, University of California, San Francisco.

[16] J.A. Maier, C. Martinez, K. Kasavajhala, L. Wickstrom, K.E. Hauser and C. Simmerling. J. Chem. Theory Comput. , 2015, 11, 3696-3713.
